# DIFFERENT ASSOCIATIONS BETWEEN HEART RATE VARIABILITY AND EMOTION REGULATION IN OLDER AND YOUNGER ADULTS

**DOI:** 10.1093/geroni/igad104.0217

**Published:** 2023-12-21

**Authors:** Kathy Liu

**Affiliations:** University College London, London, England, United Kingdom

## Abstract

Heart rate variability (HRV), the beat-to-beat variation in heart rate, is proposed to index aspects of self-regulatory processes such as emotion regulation. This is due to overlapping neural regions and networks that contribute to the central autonomic and emotion regulation systems. Paradoxically, increasing age is associated with both improvement in emotion regulation and decline in HRV, and it is unclear whether and how the association between HRV and emotion regulation is influenced by aging. The earliest stages of neurodegenerative processes, such as Alzheimer’s disease-related reduction of locus coeruleus (LC) integrity, may also influence the HRV-emotion regulation relationship in older adults, as LC modulates arousal and autonomic activity in response to stress. We pre-registered a study to investigate the cross-sectional relationship between measures of emotion regulation, HRV and LC MRI integrity in a lifespan sample of cognitively normal healthy adults using the Cambridge Centre for Ageing and Neuroscience (Cam-CAN) dataset, a large population-based cohort aged between 18-88 years (N=678). We hypothesized that any age-related differences in the association between HRV and emotion regulation would be related to reduced LC integrity in older versus younger adults. We found an inverse correlation between resting HRV and measures of emotional reappraisal in older adults, which differed significantly to younger adults, but no evidence of LC influence on this association. Our findings highlight age-related differences in the HRV-emotion regulation relationship which warrant further investigation in future studies.

